# Over Two Years of Sustained Remission With Olaparib Monotherapy in Stage IV Non‐Small Cell Lung Cancer With ATM Mutation: A Case Report

**DOI:** 10.1002/cnr2.70276

**Published:** 2025-07-07

**Authors:** Zhiting Tang, Ran Bi, Mutasim Idriss, Qian Wang, Qi Wang, Makiko Ban‐Hoefen

**Affiliations:** ^1^ Department of Medicine Unity Hospital, Rochester Regional Health Rochester New York USA; ^2^ Department of Internal Medicine Inspira Medical Center Vineland Vineland New Jersey USA; ^3^ Division of Oncology, Department of Medicine University Hospitals Seidman Cancer Center, Case Comprehensive Cancer Center, Case Western Reserve University Cleveland Ohio USA; ^4^ Department of Medicine, MetroHealth Medical Center Case Western Reserve University Cleveland Ohio USA; ^5^ Division of Hematology and Oncology, Department of Medicine University of Rochester Medical Center Rochester New York USA

**Keywords:** ATM mutation, homologous recombination deficiency, NSCLC, olaparib

## Abstract

**Background:**

Ataxia telangiectasia mutated (ATM) mutations represent the most common homologous recombination deficiency (HRD) mutation in non‐small cell lung cancer (NSCLC). However, their therapeutic role in NSCLC has not been established.

**Case:**

Here, we present a case of a 91‐year‐old male with metastatic NSCLC who progressed on multiple lines of treatment. Next‐generation sequencing revealed ATM mutations, leading to the initiation of olaparib, which successfully achieved remission over a two‐year period.

**Conclusion:**

This case underscores the promising role of olaparib in treating NSCLC with HRD, particularly ATM mutations, highlighting the importance of molecular testing and targeted therapies.

## 1 Introduction

Lung cancer is the leading cause of cancer‐related mortality worldwide. In the United States, an estimated 234 580 new lung cancer cases and 125 070 deaths are predicted in 2024 [1]. Non‐small cell lung cancer (NSCLC) represents 80% of all lung cancer cases [1]. While significant strides have been made in targeted therapy and immunotherapy in NSCLC, with a yearly decline in death rates of 5.9%, the five‐year survival rate for advanced‐stage patients remains low at 9% [2, 3].

For patients with advanced NSCLC, in addition to patients' performance status, the treatment paradigm is largely driven by the tumor programmed death ligand 1 (PD‐L1) expression and molecular testing for actionable mutations. Approximately 60%–80% of patients with advanced NSCLC will be identified to have a driver alteration that is potentially actionable [4]. At the time of this publication, the following genetic driver alterations have available targeted agents, including Epidermal Growth Factor Receptor (EGFR), Anaplastic Lymphoma Kinase (ALK) rearrangements, KRAS G12C, ROS1, BRAF V600E, NTRK 1/2/3, MET exon 14 skipping mutation, RET, and ERBB2 (HER2) [4]. Patients without actionable mutations are usually treated with immunotherapy +/− platinum‐based chemotherapy as first‐line treatment and have limited second‐line options. However, the treatment response can vary by the type and co‐occurrence of non‐actionable mutations [5].

Ataxia telangiectasia mutated (ATM), a protein kinase encoded by the ATM gene, plays a vital role in double strand DNA repair through homologous recombination [6]. Pathogenic germline variants in the ATM gene are characteristic of the inherited disorder ataxia telangiectasia (A‐T), which confers a lifelong predisposition to breast, ovarian, prostate, and pancreatic cancers [7]. Somatic ATM mutations are also seen across various cancer types and are frequently tested through NGS. In NSCLC, ATM mutations represent the most common homologous recombination deficiency (HRD) mutation, occurring in approximately 9%–11% of cases [8, 9]. However, their therapeutic implications in NSCLC have not been established. Here, to our knowledge, we present the first case of stage IV NSCLC with ATM mutation achieved sustained remission on olaparib monotherapy.

### 1.1 Case

A 91‐year‐old male with a complex medical history, former smoker, was diagnosed with liver metastases from cancer of unknown primary in September 2014 at University of Rochester Medical Center. Liver biopsy revealed poorly differentiated carcinoma which is positive for CK7 and CEA, and negative for CK20, Napsin A, Synaptophysin, PSA, PAX8, and TTF‐1, suggesting potential primary sites including lung, upper GI tract, pancreatic, or biliary tree. To further investigate, he underwent two repeat PET‐CT scans and an extensive GI workup, including EGD and colonoscopy, which were all unrevealing. Additionally, his tumor markers (AFP, CA 19–9, CEA, and PSA) were also negative. Based on these findings, a lung primary was deemed the most plausible diagnosis. The patient was initially treated with a weekly dose reduced paclitaxel (40 mg/m^2^) and carboplatin (area under the curve [AUC] of 2), along with radiation therapy targeting the liver lesion. However, a restaging scan showed a new lesion in the left pubic bone which was identified as adenocarcinoma via biopsy. Second and third‐line therapies were given, including gemcitabine (800 mg/m^2^ weekly) with nab‐paclitaxel (80 mg/m^2^ weekly) and nivolumab (3 mg/kg every 2 weeks). Unfortunately, restaging PET‐CT indicated worsening liver lesions.

His liver lesions remained stable from 2016 to 2019 with subsequent irinotecan therapy (80 mg/m^2^, 3 weeks on, 1 week off). However, new bilateral pulmonary nodules emerged in 2017, which have since increased in number and size. Meanwhile, his functional status declined significantly, resulting in multiple hospitalizations, including an admission to Thompson Hospital in June 2019 for acute kidney injury, followed by an admission to Strong Memorial Hospital in January 2021 for vomiting and dehydration. A watchful waiting approach for his pulmonary lesions was adopted.

In October 2021, PET‐CT revealed a large volume of pleural effusion and increased mildly hypermetabolic pulmonary nodules. Circulating cell‐free DNA (cfDNA) was sent and identified an ATM R3008C mutation and an ATM L427S mutation with variant allele frequencies (VAF) of 1.1% and 0.6%, respectively. Additional mutations identified included FGFR3 A391A (VAF = 0.5%) and CHEK2 M1 (VAF = 0.4%). Common NSCLC oncogenic driver mutations, including EGFR, ALK, KRAS, BRAF, and others, were tested negative. The discovery of ATM mutations prompted the consideration of olaparib therapy, a poly‐ADP ribose polymerase inhibitor (PARPi). Although olaparib has been used as a targeted therapy for ATM‐mutated ovarian and prostate cancers, its application in treating ATM‐mutated NSCLC has not been established. However, considering the patient's poor functional status and disease progression following multiple lines of therapy, this appeared to be the only viable therapeutic option. Despite the challenging circumstances, the patient expressed a strong determination to pursue further cancer‐directed therapy. Therefore, a shared decision was made to initiate a trial of olaparib.

**FIGURE 1:**
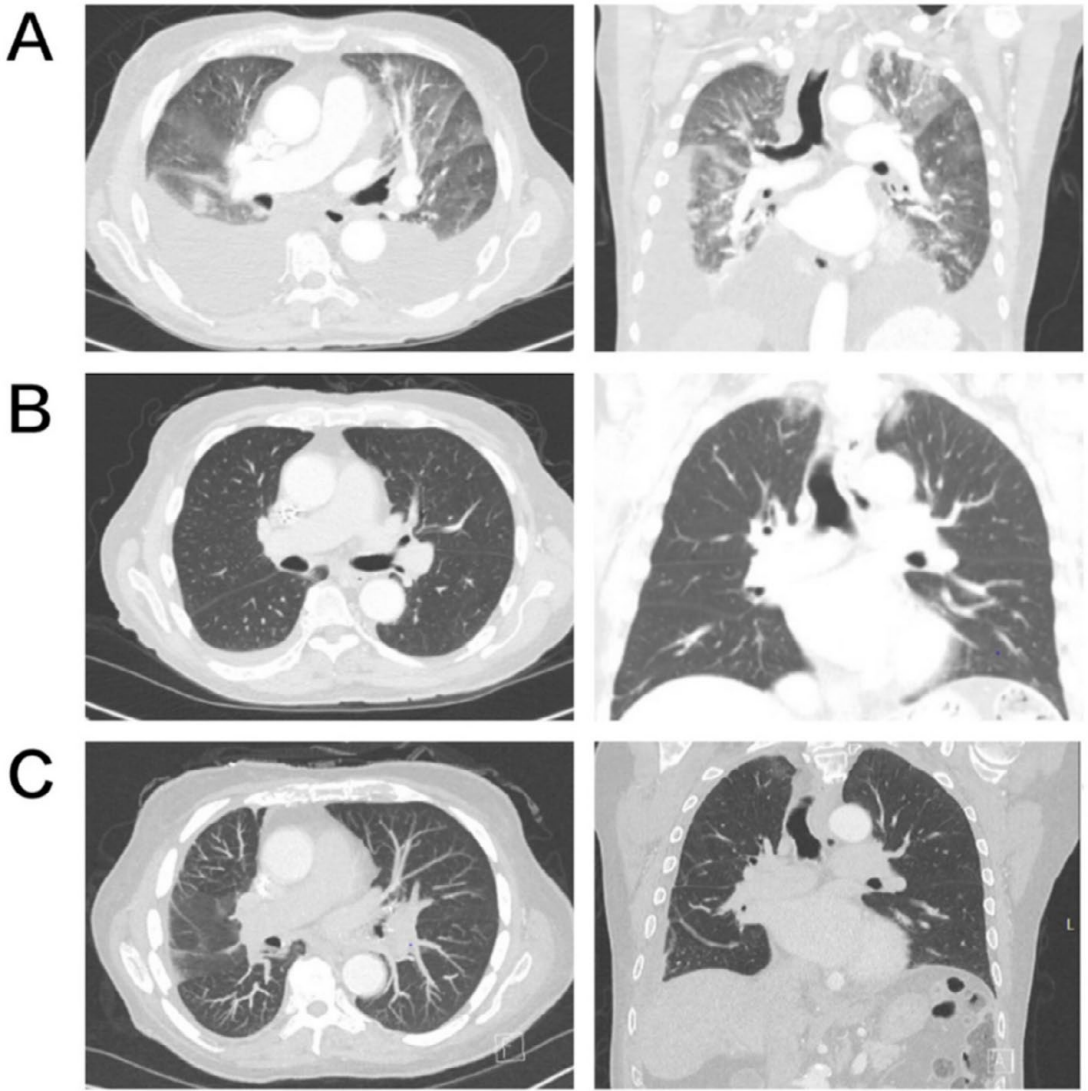
Radiographic response to olaparib on CT scans. (A) CT chest with contrast before olaparib revealed bilateral consolidative opacities in the left apex, right middle lobe, and right lower lobe, together with moderate‐to‐large pleural effusions. (B) Surveillance CT obtained 7 months after initiating olaparib demonstrated significant interval decrease in pleural effusions and resolution of the areas of consolidation within the left apex, right middle lobe, and right lower lobe. (C) Eighteen‐months follow‐up CT showed further diminution of the residual right pleural effusion, with no evidence of recurrent parenchymal disease.

**FIGURE 2:**
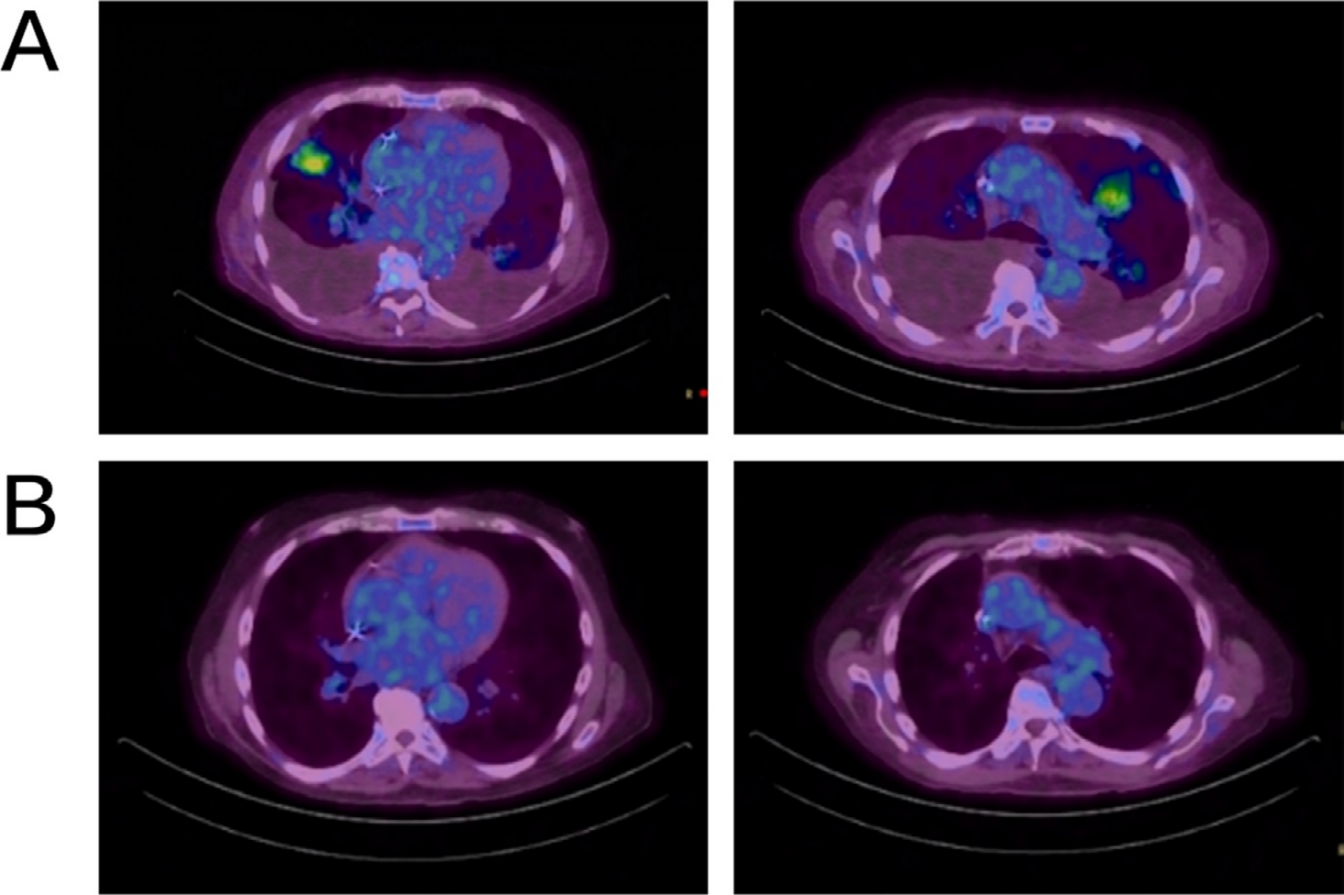
Radiographic response to Olaparib on PET‐CT scans. (A) PET‐CT in October 2021 prior to olaparib therapy showed multiple new hypermetabolic ill‐defined bilateral upper lobe opacities, including a 3.0 × 2.9 cm lobular left upper lobe lung mass (SUV max 5) and a 3.9 × 2.6 cm lobulated right upper lobe lung mass (SUV max 6). (B) Follow up PET‐CT in December 2023 after olaparib therapy demonstrated resolution of the previously noted pulmonary lesion.

**FIGURE 3:**
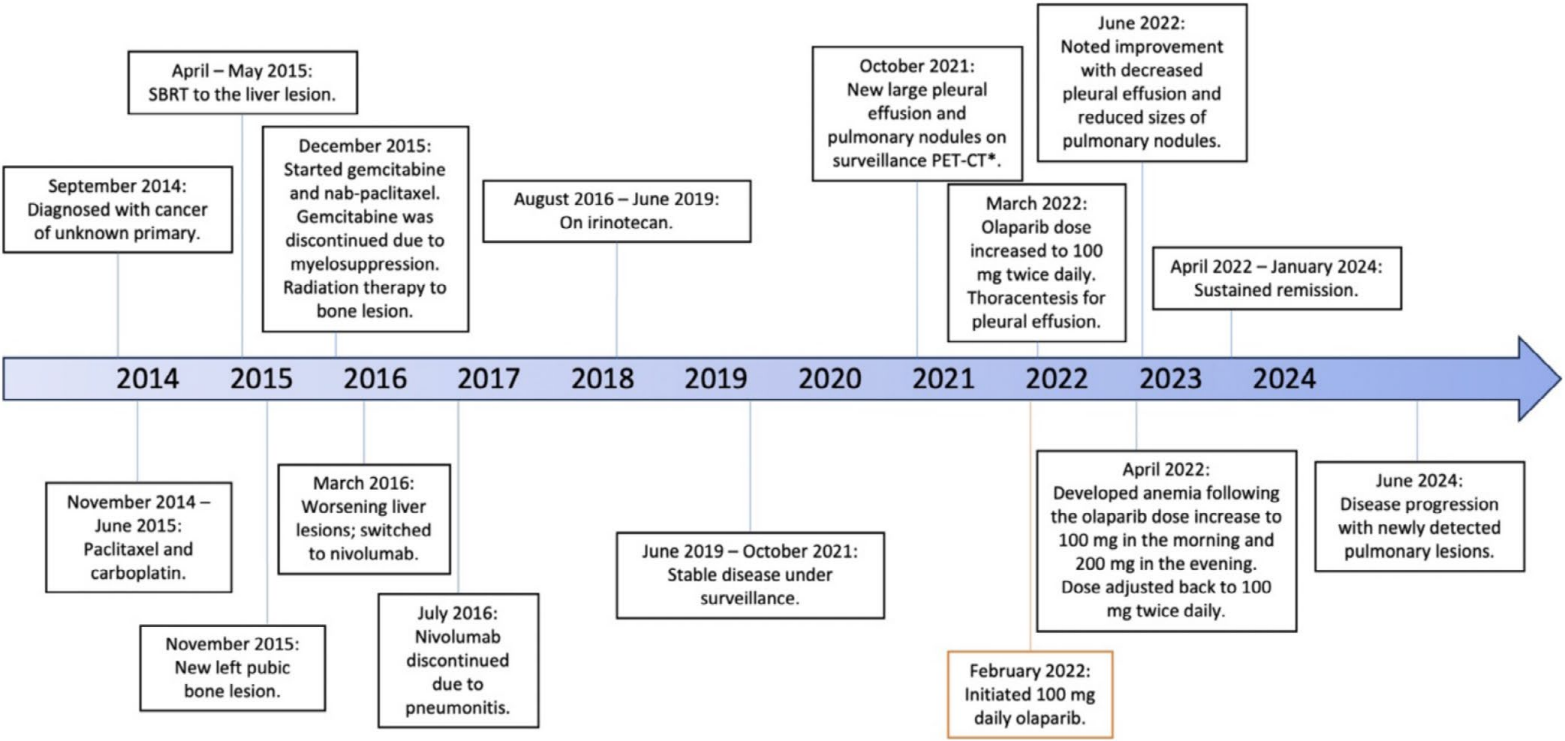
Treatment Timeline. Although initially diagnosed as cancer of unknown primary, the evolving clinical and radiologic findings supported lung cancer as the presumed origin.

In February 2022, olaparib was initiated at a dose of 100 mg daily and subsequently escalated to 100 mg twice daily. Further dose escalation was halted due to worsening anemia. Subsequent scanning at 7, 18, and 22 months demonstrated sustained disease remission with a near‐complete radiologic response (Figures 1 and 2). The latest PET‐CT in June 2024 showed disease progression with a new 1.2 cm hypermetabolic left lower lobe pulmonary nodule; nevertheless, the patient had achieved 28 months of remission and improved quality of life, notably freedom from pleurocentesis, with olaparib therapy (Figure 3 treatment timeline). At the last clinic visit in October 2024, olaparib was continued and repeat imaging was scheduled, although the patient has since experienced intermittent hospitalizations.

## 2 Discussion

Ataxia telangiectasia mutated (ATM) is a protein kinase encoded by the ATM gene located at 11q22‐23, crucial for cell cycle control, DNA double‐strand break repair, and telomere length maintenance [6]. Unlike the germline ATM mutations seen in ataxia‐telangiectasia (A‐T), which typically present as truncating/deleterious mutations, somatic ATM mutations exhibit a broader range, including various missense mutations with variable clinical significance [9]. In our patient, two ATM mutations were detected: ATM R3008C and ATM L427S mutations. ATM R3008C is recognized as an oncogenic loss‐of‐function missense mutation affecting the phosphatidylinositol‐3 kinase (PI‐3 K) domain of ATM, corroborated by preclinical studies [10, 11, 12]. However, the significance of the ATM L427S missense mutation remains unclear, necessitating further research to determine whether it is a driver mutation or merely a coincidental finding. It is important to mention that ATM mutations, along with other DNA repair genes, are frequently found in clonal hematopoiesis of indeterminate potential (CHIP), which can confound cfDNA test results [13]. A study found that 10% of advanced prostate cancer patients had CHIP interference in DNA repair genes on cfDNA tests [13]. In our case, the patient's significant clinical response to olaparib suggests that the ATM mutation originated from NSCLC rather than CHIP. Interestingly, he also had a prolonged response to irinotecan, which may further suggest that the ATM mutation originated from his cancer. Irinotecan induces single‐ and double‐strand DNA breaks and has demonstrated sensitivity in ATM‐mutated tumors, as well as a synergistic effect with PARP inhibitors in preclinical studies [14, 15].

As the most frequently detected HRD mutation in NSCLC, ATM mutation has drawn considerable attention in NSCLC research. Recent retrospective analyses have identified ATM mutations as more prevalent among female, smoker, and non‐squamous NSCLC patients. Co‐mutations in KRAS and a high tumor mutation burden (TMB) are frequently observed [8, 9]. These studies have shown conflicting results in terms of the response to immunotherapy alone versus combined immunotherapy–chemotherapy, and the role of targeted therapy remains unexplored.

Olaparib, a poly (ADP‐ribose) polymerase inhibitor (PARPi), has emerged as a promising therapeutic agent in cancers harboring HRD mutations. It induces cancer cell death by disrupting DNA single‐strand break repair, promoting the conversion of single‐strand breaks to cytotoxic double‐strand breaks, which are typically repaired in homologous recombination proficient cells but remain unresolved in HRD [16]. Originally developed to target BRCA mutations, Olaparib's indication has expanded to include other HRD mutations. It has been approved by the FDA as subsequent line therapy in ovarian cancer and prostate cancer with HRD, independent of BRCA mutation status.

Preclinical studies have shown that NSCLC cells with ATM mutations are hypersensitive to olaparib [17]. However, two recent randomized controlled trials failed to show positive results. The phase III KEYLYNK‐012 study, investigating concurrent chemoradiotherapy plus pembrolizumab with or without olaparib in stage III NSCLC patients, was discontinued in 2023 due to interim analysis failing to demonstrate an overall survival benefit [18]. Similarly, a phase II trial in Europe investigating PARPi as maintenance therapy for advanced NSCLC failed to show improvement in survival [19]. It is noteworthy that both trials included NSCLC patients regardless of their HRD status, which may have contributed to their lack of success. Furthermore, it is worth noting that ATM mutations have shown variable sensitivity to PARP inhibitors in preclinical studies using prostate cancer models [20, 21]. Combination strategies involving ataxia‐telangiectasia and Rad3‐related (ATR) kinase inhibitors have demonstrated greater efficacy [21].

As observed in our patient, anemia is the most prevalent hematologic side effect associated with Olaparib. Other commonly reported side effects include thrombocytopenia, fatigue, headache, dizziness, and gastrointestinal symptoms. Approximately 23% of patients will require dose modification due to these adverse events [22]. Treatment interruption becomes necessary when hemoglobin levels drop below 8, and if symptomatic anemia is present, a reduced dosage is resumed following recovery.

Given the patient's advanced age and multiple comorbidities, our clinical approach had certain limitations. Tissue biopsy of the pulmonary lesions for NGS was not pursued due to procedural risks. Although tissue analysis might have yielded more comprehensive genomic data, cfDNA testing provided a safer, non‐invasive alternative suitable for his condition. Additionally, cytogenetic data from the initial liver lesion was unavailable, limiting our ability to track molecular evolution over time. Lastly, while olaparib is not FDA‐approved for ATM‐mutated NSCLC, we initiated treatment based on preclinical evidence and extrapolation from other ATM‐mutated cancers. The drug's oral formulation made it especially convenient for this elderly patient, and it was a more tolerable therapeutic option compared to traditional cytotoxic chemotherapy, contributing to the improvement in his quality of life.

In summary, we reported a case of stage IV NSCLC with an ATM mutation achieving sustained remission on olaparib. This case highlights the promising role of PARPi in treating NSCLC with HRD, specifically ATM mutations. Further investigation into the clinical significance of different ATM mutation subtypes and other HRD genes is warranted to guide future therapeutic strategies and research design.

### 2.1 Clinical Significance

ATM is the most common homologous‐recombination deficiency in NSCLC, occurring in approximately 9%–11% of cases, yet an effective targeted therapy has not been established [6]. Although the PARP inhibitor olaparib is approved for ATM‐mutated ovarian and prostate cancers, its effectiveness in lung cancer remains largely unexplored [23]. We describe a metastatic NSCLC case harboring an ATM R3008C mutation that progressed through multiple lines of therapy and subsequently achieved durable remission with olaparib. This observation suggests PARP inhibitor merits further investigation as a potential treatment option for ATM‐mutated NSCLC.

## Author Contributions

**Zhiting Tang:** writing – original draft: led the writing of the background, case presentation, and discussion sections. **Ran Bi:** writing – original draft (abstract; background on somatic vs. germline ATM mutations and PARP inhibitors). **Mutasim Idriss:** writing – original draft (case presentation, prior therapies); visualization – initial treatment timeline. **Qian Wang:** writing – review and editing (molecular‐therapy background); writing – review and editing: (clarity of case presentation). **Qi Wang:** writing – review and editing (abstract refinement); visualization – final treatment timeline. **Makiko Ban‐Hoefen:** conceptualization – provided the primary conceptual framework for the manuscript; supervision – oversaw the project; case handling – clinical management of the case. writing – review and editing: reviewed and edited the manuscript, providing feedback on organization and clarity; visualization – follow up imaging.

## Ethics Statement

Ethical approval was waived in accordance with institutional guidelines for case reports (Section 4) [24]. The patient and their family have been fully informed and have given their consent for this case report.

## Conflicts of Interest

The authors declare no conflicts of interest.

## Data Availability

Data sharing not applicable to this article as no datasets were generated or analysed during the current study.
